# Impact of a Cancer Diagnosis on Quality of Life among Cancer Survivors and their Partners

**DOI:** 10.1093/eurpub/ckac131.148

**Published:** 2022-10-25

**Authors:** F Bessa, S Morais, L Lopes-Conceição, AR Costa

**Affiliations:** 1 EPIUnit, Instituto de Saúde Pública da Universidade do Porto, Porto, Portugal; 2 ITR, Laboratório para a Investigação Integrativa e Translacional em Saúde Populacional, Porto, Portugal; 3 Departamento de Ciências da Saúde Pública e Forenses, e Educação Médica, Porto, Portugal

## Abstract

**Background:**

Cancer survivors (CS) often experience physical, psychological and socioeconomic problems, which may have a negative effect on their quality of life (QoL). Additionally, cancer may also have a significant impact on patientś families, particularly their partners (PCS), who are typically the main informal caregiver.

**Objective:**

To estimate the association between a cancer diagnosis and a poor QoL among both members of the couples, according to sociodemographic, health-related and cancer characteristics.

**Methods:**

This cross-sectional study was based on data from the Sixth Wave of the Survey of Health, Ageing and Retirement in Europe - SHARE, conducted in 2015, in 18 countries. All cancer survivors (n = 2,040) who lived with a partner in the same household, as well as PCS (n = 2,040) were selected, and were country-, sex-, age- and education-matched (1:1) to non-cancer individuals (NC) and their partners (PNC), respectively. QoL was assessed using the Control, Autonomy, Self-Realization and Pleasure scale (CASP-12). The association between a cancer diagnosis and a poor QoL among both members of the couple was estimated through odds ratios (ORs) and 95% confidence intervals (95%CIs).

**Results:**

In nearly one-fifth of couples, both members reported a poor QoL (17.0%); this outcome was more frequently observed among CS than NC (OR = 1.31, 95%CI: 1.10-1.56). A tendency towards stronger odds of poor QoL among both couples’ members was observed among CS who lived in urban areas (OR = 1.91, 95%CI: 1.30-2.80), with no multimorbidity (OR = 2.07, 95%CI: 1.14-3.76), as well as among those diagnosed <5 years (OR = 1.65, 95%CI: 1.21-2.24) and with cancers with a usually poor prognosis (OR = 1.82, 95%CI: 1.04-3.18), when compared with respective NC.

**Conclusions:**

A poor QoL among both couples’ members was more frequent among couples dealing with cancer than those without cancer, which highlights the importance of closely monitoring CS and their partners, throughout the cancer survivorship course.

**Key messages:**

• Couples dealing with cancer more frequently reported a worse quality of life among both members, when compared with couples without cancer.

• The findings of this study highlights the importance of family-focused care from an early phase after the cancer diagnosis and throughout the cancer survivorship trajectory.

